# Identification and characterization of a new geminivirus from soybean plants and determination of V2 as a pathogenicity factor and silencing suppressor

**DOI:** 10.1186/s12870-022-03745-z

**Published:** 2022-07-22

**Authors:** Qinglun Li, Yuyang Zhang, Weiguo Lu, Xiaoyu Han, Lingling Yang, Yajuan Shi, Honglian Li, Linlin Chen, Yiqing Liu, Xue Yang, Yan Shi

**Affiliations:** 1 College of Plant Protection, Henan Agricultural University, Zhengzhou, 450002 China; 2 Institute of Crops Molecular Breeding, Henan Academy of Agricultural Sciences/ National Centre for Plant Breeding, Zhengzhou, 450002 China; 3 Guangdong Baiyun University, Guangzhou, 510550 China

**Keywords:** Soybean geminivirus A (SGVA), V2 protein, Pathogenicity, Genetic variability, RNA silencing suppressor

## Abstract

**Background:**

Soybean is one of the four major crops in China. The occurrence of viruses in soybean causes significant economic losses.

**Results:**

In this study, the soybean leaves from stay-green plants showing crinkle were collected for metatranscriptomic sequencing. A novel geminivirus, tentatively named soybean geminivirus A (SGVA), was identified in soybean stay-green plants. Sequence analysis of the full-length SGVA genome revealed a genome of 2762 nucleotides that contain six open reading frames. Phylogenetic analyses revealed that SGVA was located adjacent to the clade of begomoviruses in both the full genome-based and C1-based phylogenetic tree, while in the CP-based phylogenetic tree, SGVA was located adjacent to the clade of becurtoviruses. SGVA was proposed as a new recombinant geminivirus. Agroinfectious clone of SGVA was constructed. Typical systemic symptoms of curly leaves were observed at 11 dpi in *Nicotiana benthamiana* plants and severe dwarfism was observed after 3 weeks post inoculation. Expression of the SGVA encoded V2 and C1 proteins through a potato virus X (PVX) vector caused severe symptoms in *N. benthamiana*. The V2 protein inhibited local RNA silencing in co-infiltration assays in GFP transgenic 16C *N. benthamiana* plants. Further study revealed mild symptoms in *N. benthamiana* plants inoculated with SGVA-ZZ _V2-STOP_ and SGVA-ZZ _V2-3738AA_ mutants. Both the relative viral DNA and CP protein accumulation levels significantly decreased when compared with SGVA-inoculated plants.

**Conclusions:**

This work identified a new geminivirus in soybean stay-green plants and determined V2 as a pathogenicity factor and silencing suppressor.

**Supplementary Information:**

The online version contains supplementary material available at 10.1186/s12870-022-03745-z.

## Background

Soybean is one of the four major crops in China. Soybean seeds are rich in oil and protein, and therefore served as an important resource for food and industrial products. Soybean is vulnerable to infection by many viruses [1, 2]. More than 67 viruses have been reported to infect soybean crops worldwide [3]. Among them viruses in the family *Geminiviridae* have been found in soybeans such as common bean curly stunt virus (CBCSV) [4], soybean chlorotic spot virus (SoCSV) [5], mungbean yellow mosaic India virus (MYMIV) [6], african cassava mosaic virus (ACMV) [7], cowpea golden mosaic virus (CPGMV), dolichos yellow mosaic virus (DoYMV) and soybean mild mottle virus (SbMMoV) [8].

Geminiviruses are plant pathogens causing significant economic losses to many crops worldwide [9]. Currently, according to new 2020 ICTV taxonomy release the family is divided into fourteen genera [10], namely *Becurtovirus*, *Begomovirus*, *Capulavirus*, *Citlodavirus*, *Curtovirus*, *Eragrovirus*, *Grablovirus*, *Maldovirus*, *Mastrevirus*, *Mulcrilevirus*, *Opunvirus*, *Topilevirus*, *Topocuvirus*, *and Turncurtovirus*. *Begomovirus*, the largest genus within the family *Geminiviridae*, contains circular single-strand DNA, which can be either bipartite composed of both DNA-A and DNA-B components or monopartite containing a single DNA-A like component. The open reading frames (ORFs) of the DNA-A component encode six genes which are coat protein (ORF AV1/V1), movement protein (ORF AV2/V2), replication associated protein (ORF AC1/C1), transcriptional activator (ORF AC2/C2), a replication enhancer (ORF AC3/C3) and a AC4/C4 protein [11].

In the present study, we identified a new monopartite geminivirus from soybean plants in China, designated as soybean geminivirus A (SGVA). Infectious DNA clone of SGVA was constructed and inoculated into *Nicotiana benthamiana* plants via agrobacterium-mediated infiltration to show that SGVA causes disease symptoms. We tested the role of protein encoded by SGVA using PVX expression system and found that V2 and C1 are crucial for PVX symptom development and virus accumulation. We further identified V2 as the viral suppressor of RNA silencing (VSR) and as a pathogenicity factor.

## Results

### Identification and sequencing of an unknown geminivirus

**Fig. 1:**
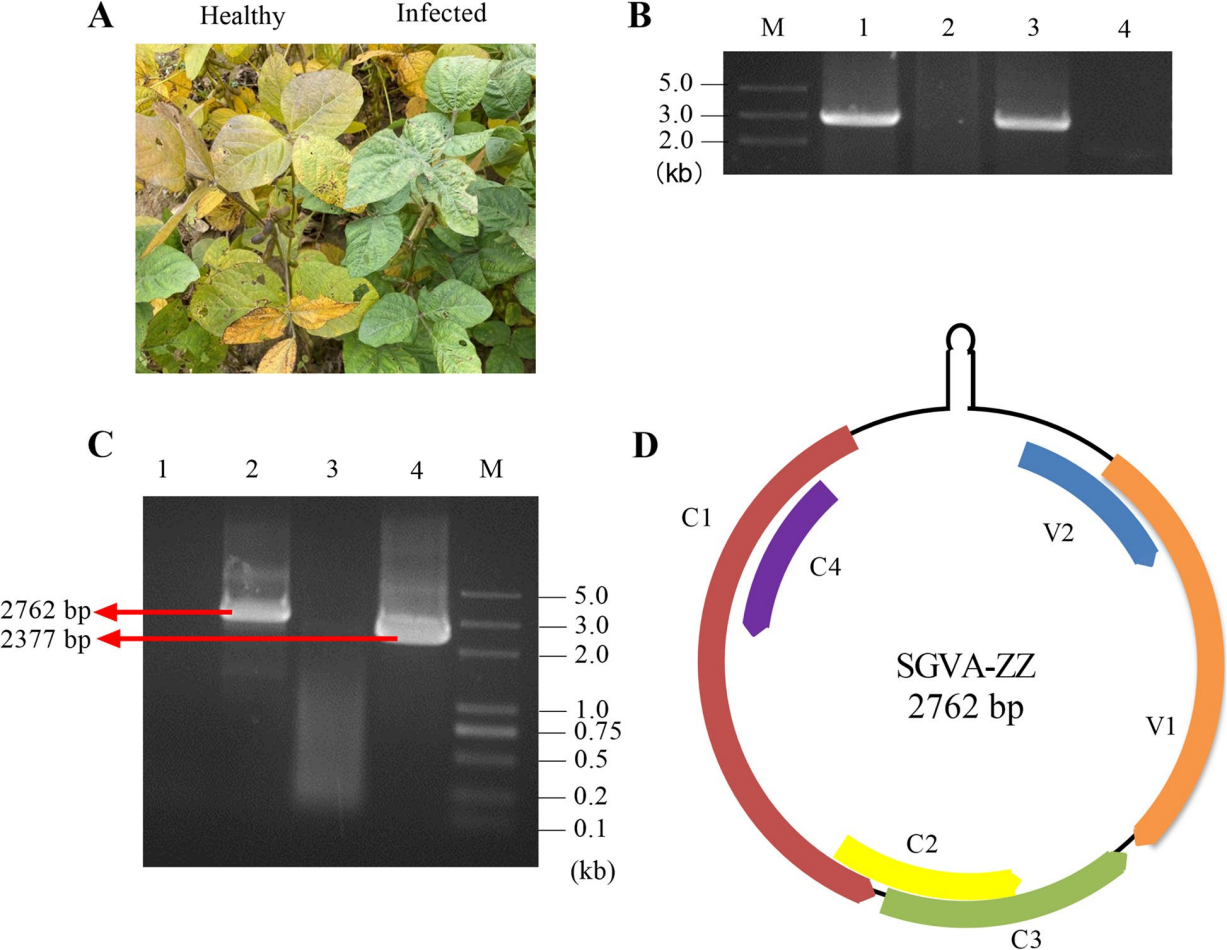
Symptoms, mRNA sequencing and detection of SGVA. **A** Symptoms of SGVA infection in the soybean stay-green plant. **B** PCR detection of the full length genome (2762 bp) of SGVA in the two field samples using primer pair AF/AR. Lane 1 and 3 are symptomatic soybean plants. Lane 2 and 4 are healthy soybean plants. **C** PCR amplification of the full length genome (2762 bp) and 0.86 copy of the genome (2377 bp). M, DNA marker. Lane 1 and 3 are healthy soybean leaves, Lane 2 and 4 are symptomatic soybean leaves. **D** Schematic diagram of the SGVA-ZZ genome

Soybean samples from Zhengzhou, China that showed crinkle and stay-green symptoms were collected (Fig. 1A) and mRNA library was constructed and sequenced using Illumina HiSeq X ten platform. In total 69,986,830 clean reads were obtained after removing low-quality reads and adaptor sequences. After assembly using the CLC Genomics Workbench (version:6.0.4), 62,243 primary unigenes were generated (Table S1). These unigenes were then assembled for a second time using CAP3 EST software to acquire the final unigene sequences. After final assembly in total 54,208 contigs were obtained (Table S2). One contig (contig7689) of 2020 nucleotides in length with the most reads of 2367 was acquired through sequencing. As shown in Fig. S1 both tomato leaf curl Cebu virus and ageratum yellow vein China virus showing the most abundant expression were mapped to the contig 7689. Using BLASTn analysis the contig showed 98.37% nucleotide identity on 97% coverage with an unpublished soybean geminivirus sequence (GenBank No. MH428829) and 83.62% nucleotide identity with tomato leaf curl Java virus (GenBank accession No. AB162141) on 55% coverage. The name “soybean geminivirus A” isolate Zhengzhou (SGVA-ZZ) is proposed for this virus. The occurrence of SGVA was detected in two symptomatic field samples (Fig. 1B). According to the sequence of contig 7689, the full-length sequence of SGVA was assembled using primer pair AF/AR and BF/BR to amplify full length of SGVA and a fragment of 2377 nt in length (Fig. 1C) and deposited in GenBank as accession No. MZ505080. The whole genome was 2762 nt in length. SGVA was found to contain six putative open reading frames (ORFs), including the V1 (786 nt) and V2 (312 nt) genes on the viral sense strand, and the C1 (1086 nt), C2 (417 nt), C3 (456 nt) and C4 (291 nt) genes on the complementary strand (Fig. 1D). A geminiviral conserved 9-base nucleotide sequence (TAATATTAC) [12] was also present in the genome of SGVA.

### Phylogenetic analysis of the viral genome

**Fig. 2:**
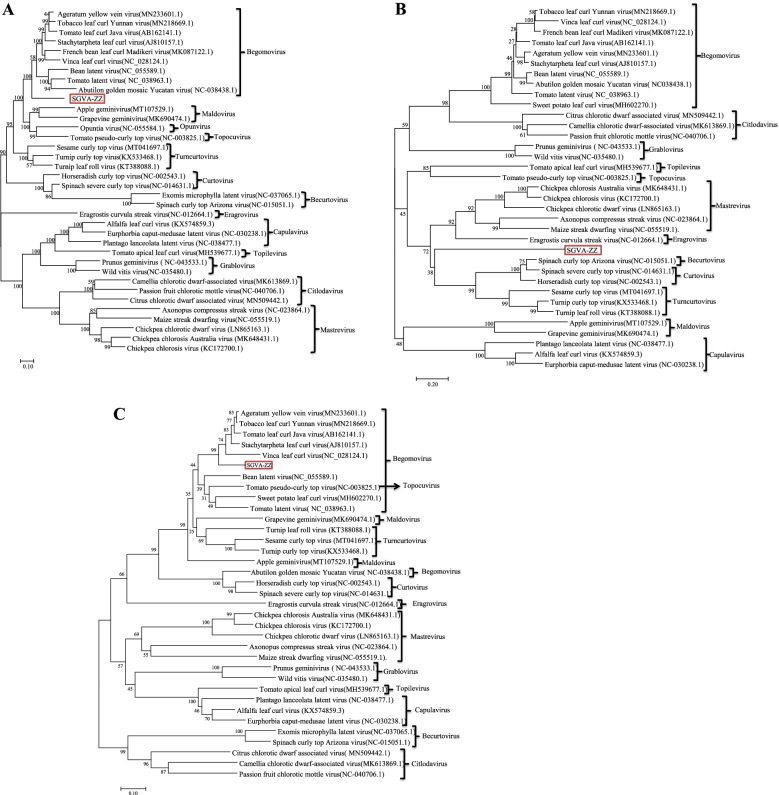
Phylogenetic analysis of SGVA. **A** Phylogenetic relationships of full genome sequences of SGVA with selected geminiviruses. The tree was constructed by the Neighbor-joining method with 1,000 bootstrap replicates in the MEGA X software. **B**, **C** Phylogenetic relationships of SGVA with selected geminiviruses based on C1 (B) and CP (C) amino acid sequences. The tree was constructed by the Neighbor-joining method with 1,000 bootstrap replicates in the MEGA X software

The neighbour-joining phylogenetic analyses based on the nucleotide sequence of the full-length genome and the amino acid sequence of coat protein (CP) (V1) and C1 were performed. SGVA-ZZ was located adjacent to the clade of begomoviruses in both the full genome-based and C1-based phylogenetic tree (Fig. 2A, B), while in the CP-based phylogenetic tree, SGVA-ZZ was located adjacent to the clade of becurtoviruses (Fig. 2C). Hence, SGVA-ZZ was proposed as a possible new recombinant geminivirus.

### Infectivity of SGVA in *Nicotiana benthamiana*

**Fig. 3:**
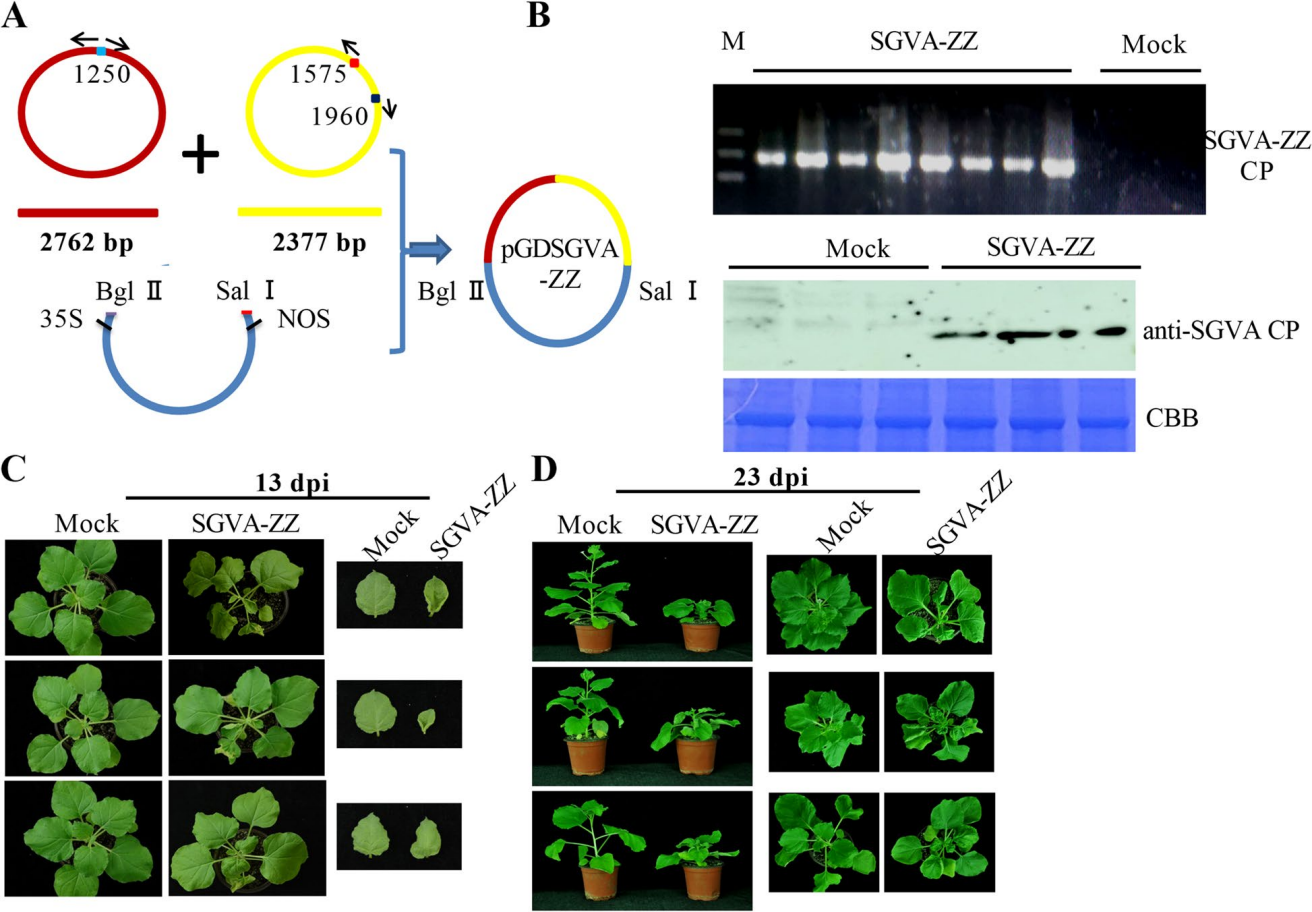
Systemic infection of SGVA-ZZ in *Nicotiana benthamiana*. **A** Construction of infectious cDNA clone. Whole viral genome was cloned using fragments F1 (2762 bp) and F2 (2377) digested with BglII/HindIII and HindIII/SalI, respectively and sequentially ligated into pGD vector to obtain the recombinant clone PGDSGVA-ZZ. **B** Systemic symptoms induced by SGVA-ZZ in *N. benthamiana* plants were observed at 13 dpi and 23 dpi, respectively. **C** The presence of SGVA-ZZ in *N. benthamiana* systemic leaves was confirmed (13 dpi) by PCR using primers specially targeting SGVA-ZZ CP. **D** SGVA accumulation in *N. benthamiana* systemic leaves was detected by conducting western blotting analysis with anti-SGVA CP antibodies. Coomassie Brilliant Blue (CBB) staining of the large subunit of RuBisCo served as a loading control

The full-length cDNA clone of SGVA was constructed into vector pGD to acquire the recombinant vector, pGDSGVA (Fig. 3A). The resultant clone was agro-infiltrated into 4-week-old leaves of *N. benthamiana* plants. Typical systemic symptoms of curly leaves were observed at 11 days post inoculation (dpi), and severe dwarfism was observed after 3 weeks post inoculation (Fig. 3B). At 33 dpi the flowering of SGVA infected *N. benthamiana* plants was inhibited compared to control plants (Fig. S2). To verify the presence of viral DNA in systemic leaves at 13 dpi, total DNA was extracted and detected using the primer pair CP-F/R (Fig. 3C). The results indicated that the infection rate was 100% for SGVA during three biological repeats of 10, 8 and 8 plants, respectively. Furthermore, SGVA accumulation in systemic leaves at 13 dpi was indicated by performing western blotting with SGVA CP polyclonal antibodies (Fig. 3D).

### Screening for potential virulence factors encoded by SGVA-ZZ

**Fig. 4:**
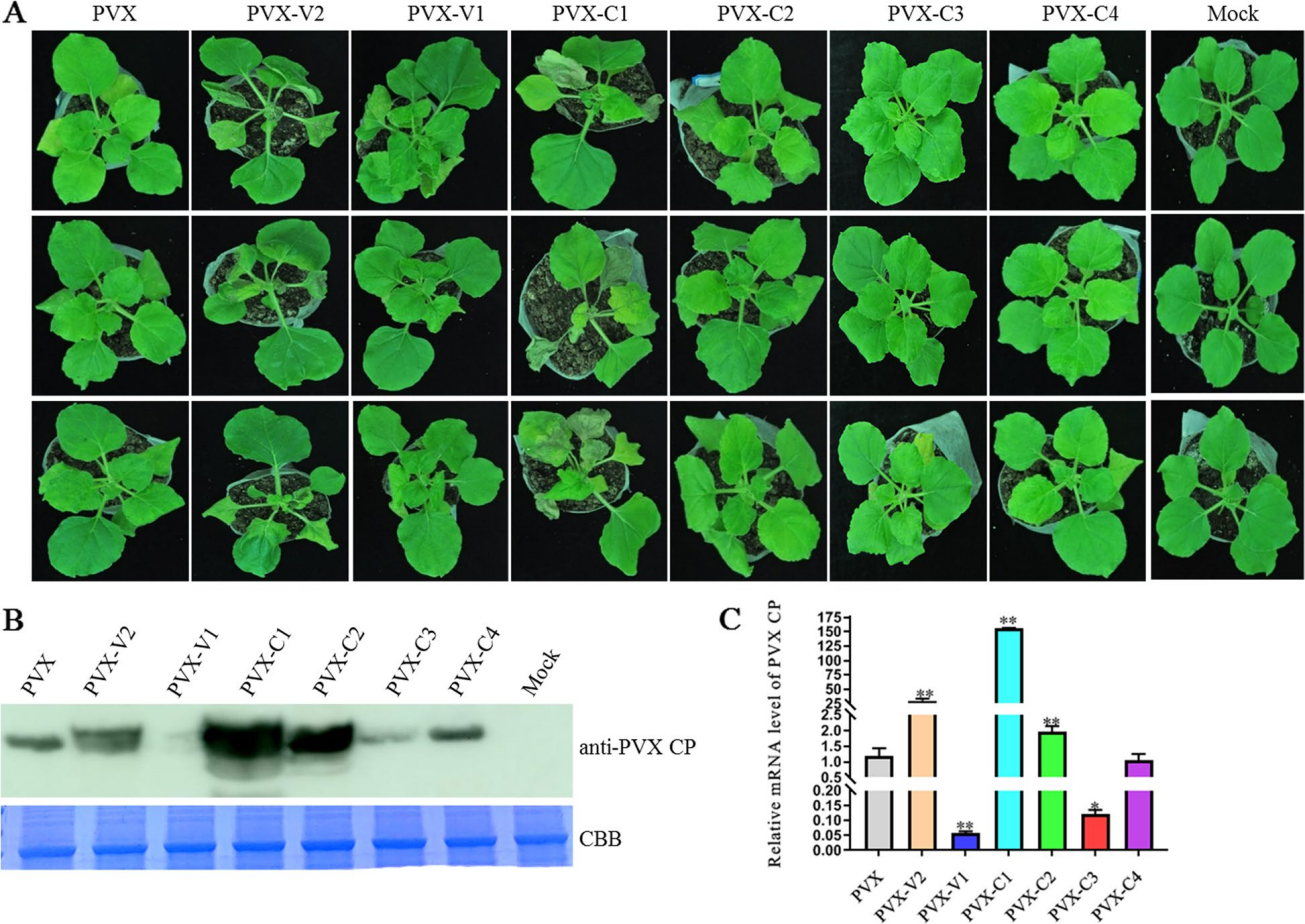
SGVA V2 and C1 enhance pathogenicity of PVX. **A** Symptoms of *N. benthamiana* plants inoculated with PVX, PVX-V1, PVX-V2, PVX-C1, PVX-C2, PVX-C3 and PVX-C4. Symptoms were photographed at 12 dpi. The accumulation of PVX was detected by western blotting (**B**) and quantitative reverse transcription PCR (qRT-PCR) (**C**) respectively. Coomassie brilliant blue-stained RuBisCo large subunit protein (CBB) was used to show sample loadings. The expression of NbUBC was used as an internal control in qRT-PCR. The results are presented as means ± SD from three biological replicates per treatment. Bars represents the mean ± standard deviation (SD). The statistical significance between treatments was determined using Duncan's multiple range test (p* < 0 .05, p** < 0.01)

To determine the potential virulence factors encoded by SGVA, six ORFs were transiently expressed in *N. benthamiana* plants via a PVX-based heterologous expression system. By 12 dpi, the *N. benthamiana* plants inoculated with PVX-V2 and PVX-C1 showed apical necrosis that ultimately led to the death of plants, and the plants inoculated with PVX-V1, PVX-C2, PVX-C3, and PVX-C4 produced PVX-like symptoms (Fig. 4A). To determine whether the severe symptoms were associated with higher virus accumulation, the expression of the CP in the systemic leaves was detected using western blotting with antibodies against PVX CP. The infection of PVX-V2, PVX-C1 and PVX-C2 promoted viral accumulation of PVX, while the accumulation of PVX-V1, PVX-C3 showed lower CP levels and the CP accumulation of PVX-C4 was equivalent to PVX (Fig. 4B). Furthermore, the viral RNA accumulation was determined using qRT-PCR (Fig. 4C), and the result was consistent with western blotting analysis.

### Identification of SGVA V2 as an RNA silencing suppressor

**Fig. 5:**
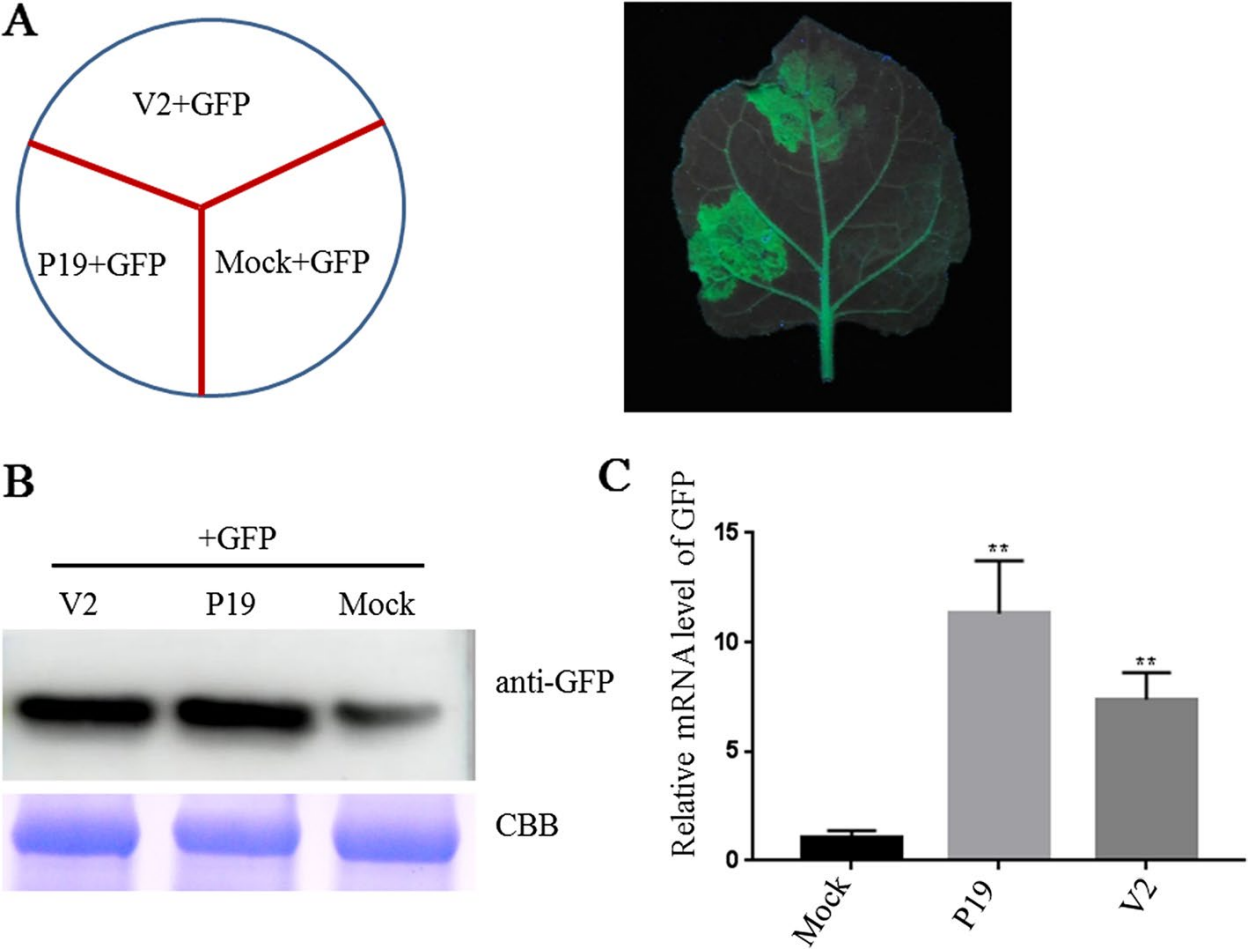
Suppression of RNA silencing by SGVA V2. **A** The leaves of GFP-transgenic 16C line were co-infiltrated with agrobacterium suspension harboring 35S-GFP expressing GFP and the recombinant vectors expressing SGVA V2 protein as indicated. The leaves expressing pGD + GFP were used as negative control and leaves expressing P19 + GFP were used as positive control. The photographs were taken under UV light at 3 d post infiltration. **B** Western blotting analysis of GFP accumulation in the co-infiltrated leaf patches at 3 d post infiltration. Coomassie brilliant blue-stained RuBisCo large subunit protein (CBB) was used to show sample loadings. **C** qRT-PCR analysis of GFP accumulation in the co-infiltrated leaf patches at 3 d post infiltration. The expression of NbUBC was used as an internal control in qRT-PCR. The results are presented as means ± SD from three biological replicates per treatment. Bars represents the mean ± standard deviation (SD). The statistical significance between treatments was determined using Duncan's multiple range test (p* < 0 .05, p** < 0.01)

V2 was previously reported as a VSR in some geminiviruses [13]. To further investigate the RNA silencing suppressor encoded by SGVA V2, ORF of V2 were introduced into the binary vector pGD. Leaves of 16C transgenic *N. benthamiana* plants carrying GFP were infiltrated with a mixture of agrobacterium harboring 35S-GFP and either a test or a control vector (Fig. 5A). The pGD + GFP agrobacterium culture harboring empty vector was infiltrated into 16C plants as negative control and P19 + GFP agrobacterium culture expressing the P19 silencing suppressor of tomato bushy stunt virus was infiltrated as positive control. At 3 d post infiltration, the leaf expressing V2 + GFP showed obvious and stronger green fluorescence under UV light similar to that produced by P19 + GFP (Fig. 5A), which correlates with the enhanced accumulation of GFP proteins by western blotting analysis and GFP mRNA by qRT-PCR analysis (Fig. 5B, C). These results suggest that SGVA V2 is an RNA silencing suppressor.

### Identification of V2 as a pathogenicity determinant

**Fig. 6:**
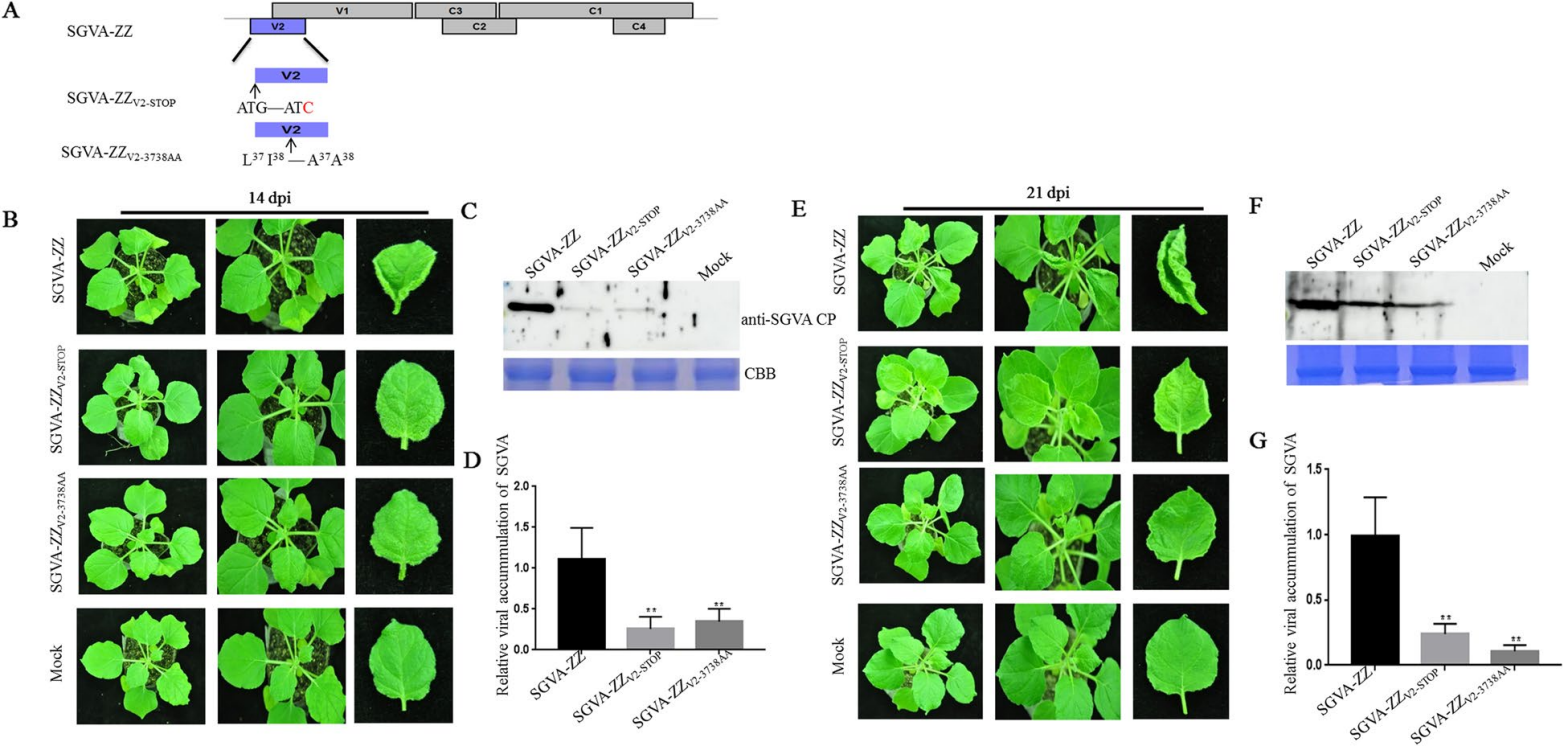
SGVA V2 functions as a pathogenicity determinant. **A** SGVA-ZZ_V2-STOP_ and SGVA-ZZ_V2-3738AA_ was constructed as shown in the schematic diagram. Symptoms of *N. benthamiana* plants inoculated with SGVA-ZZ, SGVA-ZZ_V2-STOP_ and SGVA-ZZ_V2-3738AA_ were photographed at 14 dpi and 21 dpi, respectively (**B**, **E**). The accumulation of SGVA-ZZ was detected by western blotting (**C**, **F**) and quantitative reverse transcription PCR (qRT-PCR) (**D**, **G**) respectively. Coomassie brilliant blue-stained RuBisCo large subunit protein (CBB) was used to show sample loadings. The expression of NbUBC was used as an internal control in qRT-PCR. The results are presented as means ± SD from three biological replicates per treatment. Bars represents the mean ± standard deviation (SD). The statistical significance between treatments was determined using Duncan's multiple range test (p* < 0 .05, p** < 0.01)

**Fig. 7:**
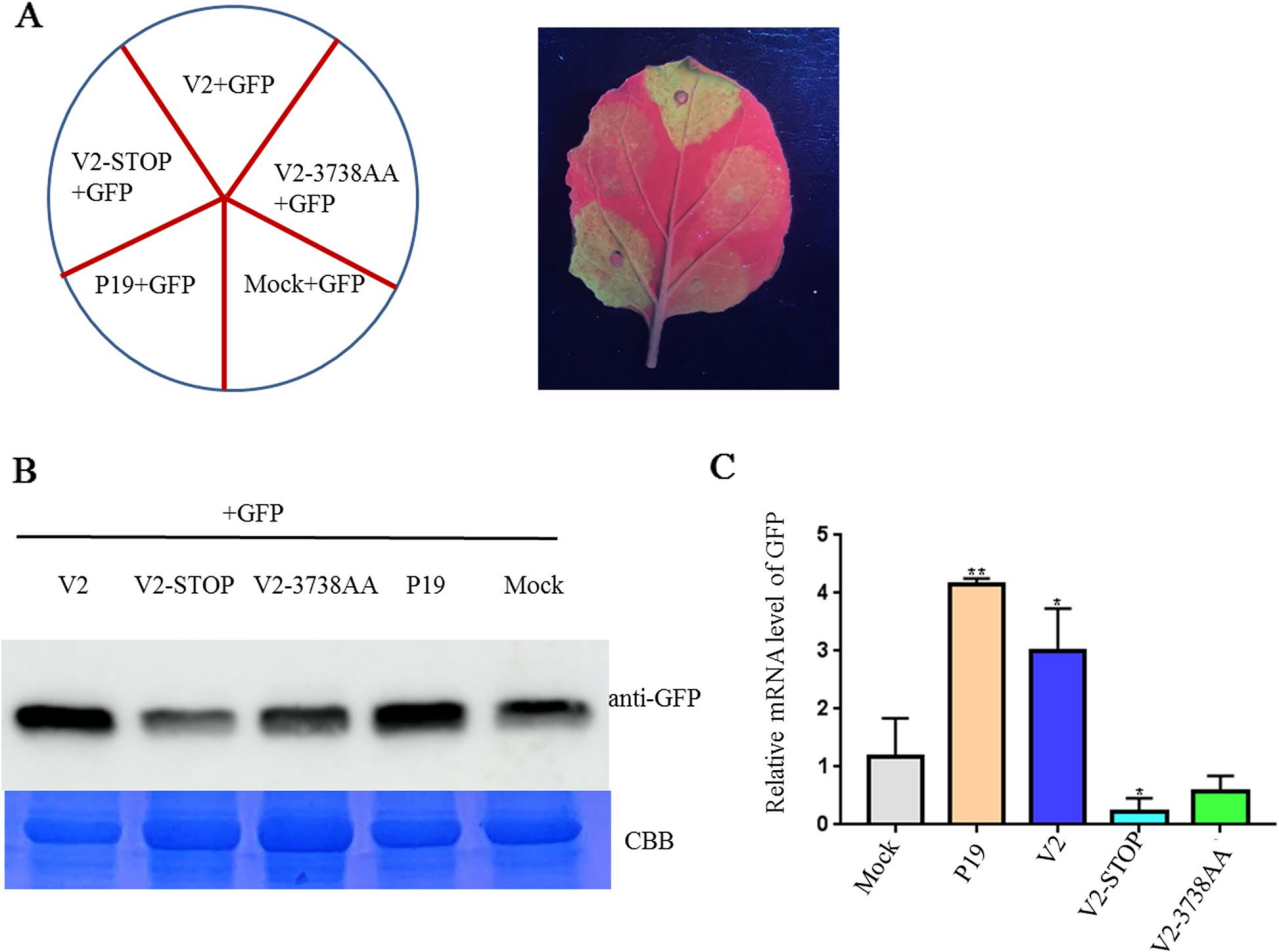
V2 mutants abolishes RNA silencing suppressor activity of V2. **A** The leaves of *Nicotiana benthamiana* were co-infiltrated with agrobacterium suspension harboring 35S-GFP expressing GFP and the recombinant vectors expressing SGVA V2, V2-STOP and V2-3738AA proteins as indicated. The leaves expressing pGD + GFP were used as negative control and leaves expressing P19 + GFP were used as positive control. The photographs were taken under UV light at 3 d post infiltration. **B** Western blotting analysis of GFP accumulation in the co-infiltrated leaf patches at 3 d post infiltration. Coomassie brilliant blue-stained RuBisCo large subunit protein (CBB) was used to show sample loadings. **C** qRT-PCR analysis of GFP accumulation in the co-infiltrated leaf patches at 3 d post infiltration. The expression of NbUBC was used as an internal control in qRT-PCR. The results are presented as means ± SD from three biological replicates per treatment. Bars represents the mean ± standard deviation (SD). The statistical significance between treatments was determined using Duncan's multiple range test (p* < 0 .05, p** < 0.01)

To further study the function of V2, we constructed two V2 mutants of SGVA-ZZ. SGVA-ZZ_V2-STOP_ mutant in which the start codons in the V2 gene (ATG) was modified to ATC. Besides we performed an alignment using the amino acid sequence of SGVA-ZZ V2 and that of other geminivirus V2 with the DNAMAN software (Fig. S3). Two conserved amino acids (L37, I38) in the V2s were found and were substituted with alanine to generate SGVA-ZZ_V2-3738AA_ mutant (Fig. 6A). Then, *N. benthamiana* were inoculated separately with SGVA-ZZ, SGVA-ZZ_V2-STOP_ and SGVA-ZZ_V2-3738AA_. At 14 dpi, the SGVA-ZZ inoculated *N. benthamiana* plants showed obvious symptoms of curly leaves, while the SGVA-ZZ_V2-STOP_ and SGVA-ZZ_V2-3738AA_ inoculated plants did not show obvious virus symptoms (Fig. 6B). Western blot and qRT-PCR analysis results showed that SGVA-ZZ viral DNA and CP accumulations were significantly reduced in the systemic leaves of the SGVA-ZZ_V2-STOP_ and SGVA-ZZ_V2-3738AA_ inoculated plants compared to that in the systemic leaves of the SGVA-ZZ inoculated plants, indicating that V2 expression was essential for SGVA-ZZ systemic infection and conserved amino acids L37I38 mutation inhibited the systemic infection of SGVA-ZZ (Fig. 6C, D). Furthermore, by 21 dpi, the SGVA-ZZ inoculated *N. benthamiana* plants showed severe symptoms, while the SGVA-ZZ_V2-STOP_ and SGVA-ZZ_V2-3738AA_ inoculated plants showed mild symptoms of curly leaves (Fig. 6E). Western blot and qRT-PCR analysis further confirmed the symptoms observed (Fig. 6F, G). Furthermore, the VSR activity of the two V2 mutants were examined in *N. benthamiana* plants. The pGD + GFP agrobacterium culture harboring empty vector was infiltrated into *N. benthamiana* plants as negative control and P19 + GFP agrobacterium culture was infiltrated as positive control. At 3 d post infiltration, the leaf expressing V2 + GFP showed obvious and stronger green fluorescence under UV light similar to that produced by P19 + GFP while V2-STOP + GFP and V2-3738AA + GFP showed weak fluorescence as pGD + GFP control (Fig. 7A). Further detection of GFP proteins and mRNA by western blotting and qRT-PCR analysis confirmed the observation (Fig. 7B, C).

## Discussion

The family Geminiviridae consists of fourteen genera that mainly affect a wide range of plants. Of all the fourteen genera, *Begomovirus* is the largest member of the group, which has about 445 species. Several begomoviruses have been reported to infect soybean [4–8]. In this study we characterized a new geminivirus named SGVA from diseased soybean stay-green plants showing leaf curling symptoms in Zhengzhou, China. Sequence analysis reveals that SGVA shares 98.37% identity with the unpublished Genbank sequences (GenBank No. MH428829). According to the phylogenetic analysis, SGVA was adjacent to the clade of begomoviruses based on full-length nucleotide sequence and C1 amino acid sequences, while SGVA was adjacent to the clade of becurtoviruses based on CP amino acid sequences. SGVA was proposed as a new recombinant geminivirus. The high genetic variability of geminivirus populations is predominantly driven by their high mutational dynamics combined with recombination [14–16].

In this study we found that the over-expression of V2 protein induced systemic necrosis in* N. benthamiana* plants, and indicating that it functions as a key virulence factor. Besides, many V2 proteins encoded by different geminiviruses have been demonstrated to be important virulence determinants [13, 17, 18]. PVX-C1-inoculated plants started to show systemic necrosis at 5 dpi. Previous research showed that PVX expressing C1 of apple geminivirus (AGV) developed visible necrotic lesions at 13 dpi [19]. To further confirm the function of V2 in viral pathogenicity, two SGVA mutants, SGVA-ZZV2-STOP and SGVA-ZZV2-3738AA, were constructed. Both mutants showed milder symptoms and lower viral accumulation compared with SGVA inoculated *Nicotiana benthamiana* plants indicating V2 functions as a pathogenicity factor during SGVA infection. The over-expression of V1 and C3 showed lower viral accumulation than PVX possibly due to the activation of host defense related pathways by V1 and C3 over-expression and thereby inhibited viral accumulation. C2 over-expression promoted PVX CP accumulation at higher level than the mRNA accumulation. C2 plays role in the suppression of host immune responses and regulation of the ubiquitin/proteasome system (UPS) [20]. It is possible that C2 interferes with the UPS pathway and promote the CP accumulation. Multiple virus encoded RNA silencing suppressors are involved in viral pathogenicity, such as pepper vein yellows virus P0, tomato leaf curl Java virus V2 protein, rice stripe mosaic virus P4 protein, watermelon silver mottle virus nonstructural protein S [18, 21–23]. In our study SGVA encoded V2 protein acts as a RNA silencing suppressor and pathogenicity determinant which is consistent with the previous studies.

Here in our study we identified a new geminivirus in soybean stay-green plants and established the infectious clone system in *Nicotiana benthamiana*. We’ve tried different inoculation methods including agro-inoculation, shot-gun and sap-inoculation to inoculate two varieties of soybean, whereas the infection was not established in soybeans plants. Considering SGVA may be a threat to soybean production in China, further efforts to elucidate the impact of SGVA on soybean plants are underway.

## Conclusions

In this work we identified a new monopartite geminivirus from soybean stay-green plants in China, designated as soybean geminivirus A (SGVA). Infectious DNA clone of SGVA was constructed and inoculated into *Nicotiana benthamiana* plants via agrobacterium-mediated infiltration to show that SGVA causes disease symptoms. We further identified V2 as the viral suppressor of RNA silencing (VSR) and as a pathogenicity factor. Conserved amino acids L37I38 of V2 are essential for the viral pathogenicity.

## Materials and methods

### Plant materials and virus inoculation

During field survey in August 2020, soybean samples exhibiting crinkle and stay-green symptoms were collected from Zhengzhou, Henan province of China. *N. benthamiana* plants were grown in pots in a growth room under a 16 h light/8 h dark photoperiod at 25 °C with 60% humidity. For agroinfiltration, Agrobacteria strain GV3101 carrying infectious viral clones were suspended in infiltration buffer (10 mM MgCl_2_, 10 mM MES, and 200 μM acetosyringone, pH 5.6) at an OD_600_ of 1, kept at room temperature for 2 to 4 h and infiltrated into *N. benthamiana* leaves using a 1-mL needleless syringe.

### Plasmid construction

Primers used for plasmid construction are listed in Table S3. All the available constructs were sequenced.

PVX recombinant vectors was constructed by introducing coding sequences of SGVA encoded proteins into potato virus X (PVX) vector pGR106 via ClaI and SalI digestion, followed by ligation with T4 DNA ligase (NEB). The fragments used were amplified using primer pairs PVX-V2/V1/C1/C2/C2/C4-F/R.

To construct vectors for RNA silencing analysis, the coding sequence of corresponding genes were amplified and inserted into PstI/BamHI digested pGD vectors via homologous recombination using Clonexpress II one step cloning kit (Vazyme, China) [24], the homologous arm length was 20 bp. The fragments used were amplified using primer pairs PGD-V2 -F/R.

To construct full-length cDNA clone of SGVA, a recombinant plasmid containing 1.8 copies of the full-length fragment of the viral genome was constructed. First, a 2762 bp full length was amplified from total DNA extracted in symptomatic leaves using Q5 high-fidelity polymerase (NEB, Beijing,China) with primer pair SGVAF1F/ SGVAF1R and ligated into binary vector pGD vector between the cauliflower mosaic virus (CaMV) 35S promoter and the nopaline synthase terminator (t-Nos) via the restriction sites BglII/ HindIII to produce construct pGDSGVAF1. After sequencing, a 2377 bp partial fragment was amplified using primer pair SGVAF2F/SGVAF2R and ligated into pGDSGVAF1 vector via the restriction sites HindIII/SalI to produce construct pGDSGVA.

To construct V2 mutant vectors, a 2762 bp full length was amplified from pGDSGVA-ZZ using primer pairs SGVAV2STOPF/ SGVAF1R, SGVAF1F/ SGVAV2STOPR and ligated into pGD vector via the restriction sites BglII/ HindIII to produce construct pGDSGVA_v2stop_F1, then a 2377 bp partial fragment was amplified using primer pair SGVAV2STOPF/ SGVAF2R, SCVF2F/ SGVAV2STOPR and ligated into pGDSGVA_v2stop_F1 vector via the restriction sites HindIII/SalI to produce construct pGDSGVA_v2stop_. SGVAV2-3738AA mutant was constructed using primer pairs SGVA3738AAF and SGVA3738AAR in the same way.

### Nucleic Acid Extraction, Metatranscriptomic Sequencing, and Data analysis

The total RNA of samples was subjected to an rRNA removal procedure using a Ribo-zero Magnetic kit according to the manufacturer’s instructions (Epicentre, an Illumina® company). Next, cDNA libraries were constructed using a TruSeq™ RNA sample prep kit (Illumina). Barcoded libraries were paired-end sequenced on an Illumina HiSeq X ten platform at Shanghai biotechnology Co., Ltd. (Shanghai, China) according to the manufacturer’s instructions (www.illumina.com).

To obtain clean reads, the Fastax online software (version: 0.0.13, http://hannonlab.cshl.edu/fastx_toolkit/index.html) was used to screen out unqualified reads from the raw reads; this step removed joint sequences, reads with low overall quality (mainly reads with lengths less than 20 bp), reads with base N (base with uncertain identity), reads with more than 20% of the bases possessing Q-values ≤ 10, and or low end quality. Then, the reads were trimmed to remove bases with a quality score of soybean. Clean reads were de novo assembled using CLC Genomics Workbench (version:6.0.4) according to the scaffolding contig algorithm (word-size = 45, minimum contig length = 300) [25–27]. These various steps produced the primary unigenes. These were then assembled for a second time using CAP3 EST software [28] to acquire the final unigene sequence set. This unigene set was used for further exploration of the transcriptome. The final unigene set was compared against the NCBI non-redundant (Nr) database using BLASTX [29], with an E-value < 1^e−5.^ Then the final unigenes annotated with virus were used for the virus analysis.The assembled contigs were queried by homology search tools (BLASTn and BLASTx) against public database (GenBank) in the NCBI (the National Center for Biotechnology Information).

### Phylogenetic analysis

Sequences alignments were conducted using the ClustalW method, and phylogenetic trees were constructed by the neighbor joining method using MEGA version X [30]. The tree was evaluated with 1000 bootstrap replicates. Sequences of geminiviruses were retrieved from GenBank and used for comparison and phylogenetic analysis in this study.

### Western blotting analysis

Agro-infiltrated leaves were harvested for western blotting assay. Total protein was extracted from 0.2 g leaves using the extraction buffer containing 20% glycerol, 20 mM Tris–HCl (pH 7.5), 1 mM EDTA, 150 mM NaCl, 1 mM PMSF, 1 × Protease inhibitor cocktail (Sigma, China). Total protein was separated in SDS–polyacrylamide gel electrophoresis, followed by transfer to nitrocellulose membranes. The membranes were probed using anti-PVX CP polyclonal antibodies or SGVA CP polyclonal antibodies followed by an HRP-conjugated secondary antibody. Antigens of PVX CP and SGVA CP was acquired via prokaryotic expression. The detection signals were developed using an ECL reagent as instructed. PVX CP and SGAV CP accumulation were photographed under a chemiluminescence apparatus (Amersham imager 680). CBB staining of the large subunit of RuBisCo served as a loading control. Due to the figure size the original size image of the blots was supplied in the supplementary information.

### Quantitative RT-PCR

Total RNA was extracted from harvested *N. benthamiana* leaves using Trizol reagent (invitrogen) and treated with RNase-free DNase I. First strand cDNA was synthesized using 500 ng total RNA, an oligo d(T) primer, random primer, and M-MLV reverse transcriptase as instructed. Ten-fold diluted cDNA product was used for qPCR on an Eppendorf Real-Time PCR system using a SYBR Green master mix (Takara). The NbUBC genes (GenBank accession number: AB026056.1) was used as internal controls. All the primers used for qRT-PCR are listed in Table S3. The relative gene expression levels were calculated using the 2^−△△CT^ method.

## Supplementary Information

**Additional file 1: Supplementary Table S1.** The statistics of primary assembly. **Supplementary Table S2.** The statistics of final assembly. **Supplementary Table S3.** List of PCR primers used in this work. **Supplementary Fig. S1.** The species expression abundance results based on the reads expression level of each species. **Supplementary Fig. S2.** Systemic symptoms induced by SGVA-ZZ in *N. benthamiana* plants were observed at 33 dpi. **Supplementary Fig. S3.** The multiple alignment of V2 amino acids of geminiviruses. **Supplementary Fig. S4**. Full scan date of the immunoblots in this work.

## Data Availability

All data generated or analyzed during this study are included in this published article and its supplementary information files.
