# Supplementary material for: Identification and characterization of a new geminivirus from soybean plants and determination of V2 as a pathogenicity factor and silencing suppressor
Source: BMC Plant Biol. 2022 Jul 22;22:362. doi: 10.1186/s12870-022-03745-z (PMC9308217; doi:10.1186/s12870-022-03745-z)
Supplement: Supplementary file 1 — Additional file 1: Supplementary Table S1. The statistics of primary assembly. Supplementary Table S2. The statistics of final assembly. Supplementary Table S3. List of PCR primers used in this work. Supplementary Fig. S1. The species expression abundance results based on the reads expression level of each species. Supplementary Fig. S2. Systemic symptoms induced by SGVA-ZZ in N. benthamiana plants were observed at 33 dpi. Supplementary Fig. S3. The multiple alignment of V2 amino acids of geminiviruses. Supplementary Fig. S4. Full scan date of the immunoblots in this work. [file 12870_2022_3745_MOESM1_ESM.docx]

**Supplementary materials**

**Supplementary Table S1.**The statistics of primary assembly.

| Statistics | counts | Total length(bp) | N25(bp) | N50(bp) | N75(bp) | Average length | longest(bp) | N% | GC% |
| --- | --- | --- | --- | --- | --- | --- | --- | --- | --- |
| contigs | 66533 | 36063630 | 1290 | 710 | 365 | 542 | 11262 | 0.2 | 42.7 |
| Primary UniGene | 62243 | 36121949 | 1394 | 773 | 390 | 580 | 11262 | 0.2 | 42.7 |

**Supplementary Table S2.** The statistics of final assembly.

| Statistics | counts | Total length(bp) | N25(bp) | N50(bp) | N75(bp) | Average length | longest(bp) | N% | GC% |
| --- | --- | --- | --- | --- | --- | --- | --- | --- | --- |
| contig | 54208 | 32359362 | 1506 | 828 | 397 | 596.95 | 11262 | 0.18 | 42.64 |

**Supplementary Table S3.** List of PCR primers used in this work.

| **Name** | **Sequence (5' - 3')** |
| --- | --- |
| AF | TCAAGGTCGTCCAGACCTTC |
| AR | CGACTGCTGGTCCGACATCC |
| BF | TCACCTTCCAGAACTATACT |
| BR | AATTATTCACAAGCAAGCCA |
| CP-F | ATGGATTACAGCAGGAAGAGG |
| CP-R | TTACAATTTGCTCTTGAAATACGTG |
| SGVAF1F | ATCACCAGTCTCTCTCTACA AGATCT TCAAGGTCGTCCAGACCTTC |
| SGVAF1R | ACCGTCGACT GCAGAATTCG AAGCTTCGAC TGCTGGTCCG ACATCC |
| SGVAF2F | CCCAAGCTT TCACCTTCCACAACTATACT |
| SGVAF2R | GCGTGTCGACAAATTATTCA CAAGCAAGCC |
| SGVAV2STOPF | TTTTCTTTATCGAGGACAGAAATATTGTCGTC |
| SGVAV2STOPR | TTCTGTCCTCGATAAAGAAAAAGACGACAGTA |
| SGVA3738AAF | TGCGGAGCAGGCAGCTCGTTGGATTAATGGATTAC |
| SGVA3738AAR | AATCCAACGA GCTGCCTGCT CCGCACTACA ACGGC |
| PVX-V2-F | AGAGGTCAGCACCAGCTAGCATCGAT ATGGA GGACA GAAAT ATTGT |
| PVX-V2-R | AACTTAACCG TTCATCGGCG GTCGACTCATTCACCTGCAG ATTGGT |
| PVX-V1-F | AGAGGTCAGCACCAGCTAGCATCGATATGGA TTACA GCAGG AAGAG |
| PVX –V1-R | AACTTAACCG TTCATCGGCG GTCGACTTACAATTTGCTCT TGAAAT |
| PVX -C1-F | AGAGGTCAGCACCAGCTAGCATCGATATGGCTCCTC CACGTCGTTT |
| PVX -C1-R | AACTTAACCG TTCATCGGCG GTCGACCTAGGTGGTTTCTT CTTGGC |
| PVX -C2-F | AGAGGTCAGCACCAGCTAGCATCGATATGCAATCTTCGTCACCATC |
| PVX -C2-R | AACTTAACCGTTCATCGGCGGTCGACCTAGATAGAACGGATGTCGG |
| PVX -C3-F | AGAGGTCAGCACCAGCTAGCATCGATATGCCTGTTGTGGCTTATGC |
| PVX -C3-R | AACTTAACCGTTCATCGGCGGTCGACTTAATATAAATTGCATTGAA |
| PVX-C4-F | AGAGGTCAGCACCAGCTAGCATCGATATGGGAGCCCTCATCTCCAT |
| PVX -C4-R | AACTTAACCGTTCATCGGCGGTCGACTCACATTAAGAGCCTCTGAC |
| PGD-V2-F | ACGATAAGAAGCTTCGAATTCTGCAGATGGAGGACAGAAATATTGT |
| PGD -V2-R | ATCAGTTATCTAGATCCGGTGGATCCTCATTCACCTGCAGATTGGT |
| qRT -PVX-CP-F | ATGTCAGCACCAGCTAGCAC |
| qRT -PVX-CP-R | TGGTGGTGGGAGAGTGACAA |
| qRT -SGVA -CP-F | AGGACCAATCTGCAGGTGAA |
| qRT -SGVA -CP-F | GCGTTCCCATTACCCAACTC |
| NbUBC-F | GAGGAAGAGACTGGTGAGGGAT |
| NbUBC-R | CACAGAGCAAAGACTGGATTGA |

**
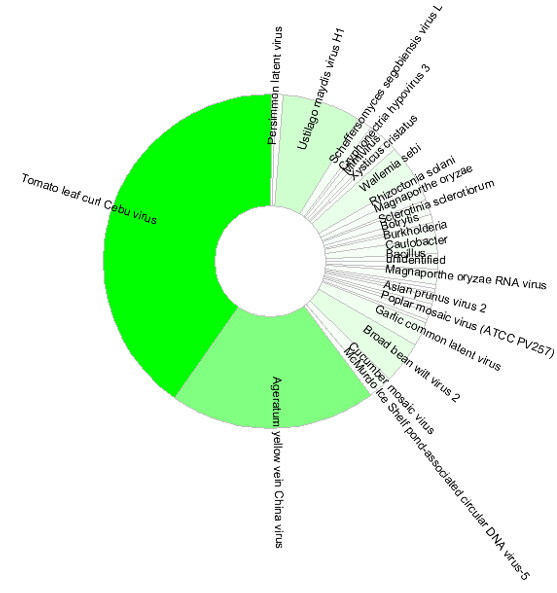
**

**Supplementary Fig. S1.** The species expression abundance results based on the reads expression level of each species.


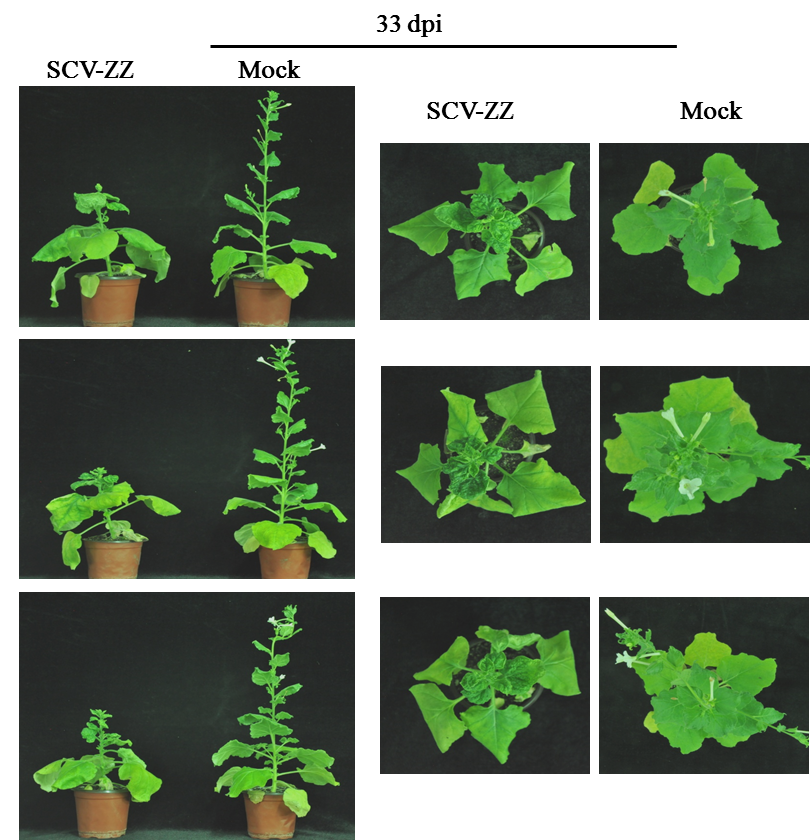


**Supplementary Fig. S2.** Systemic symptoms induced by SCV-ZZ in *N. benthamiana* plants were observed at 33 dpi.


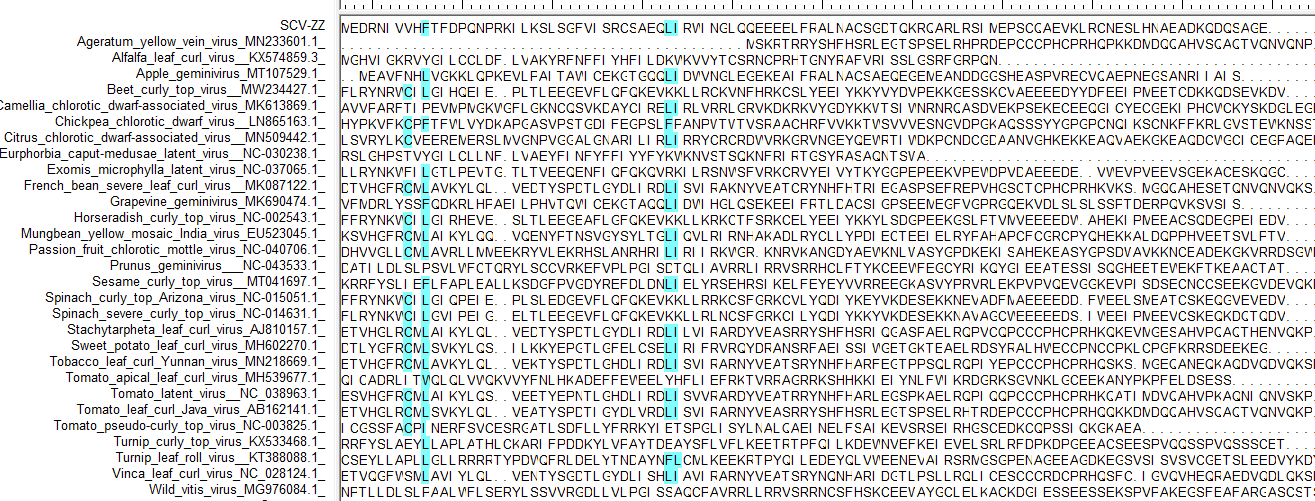


**Supplementary Fig. S3.** The multiple alignment of V2 amino acids of geminiviruses

**
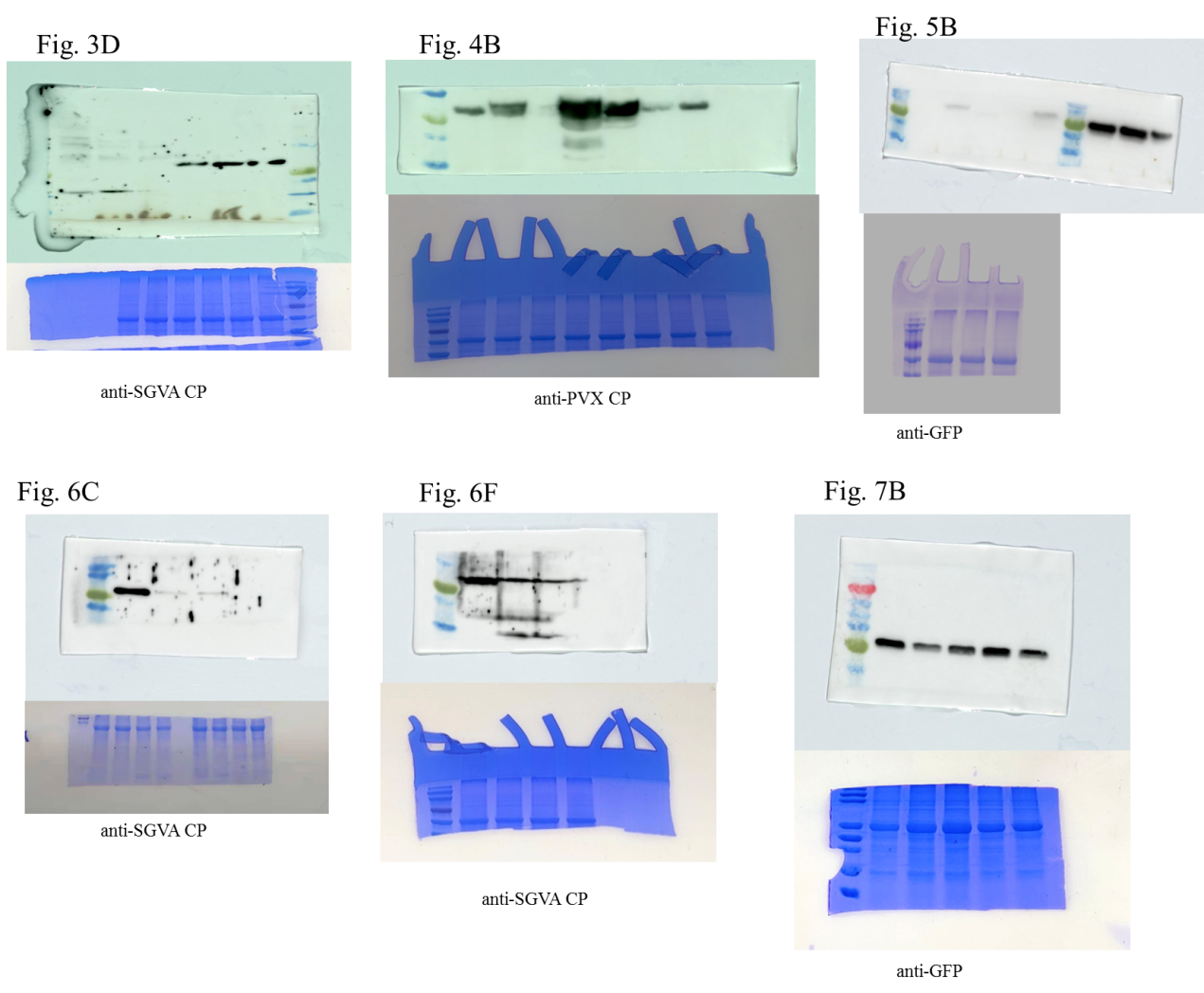
**

**Supplementary Fig. S4** Full scan date of the immunoblots in this work.
